# A Vehicle Detection Algorithm Based on Deep Belief Network

**DOI:** 10.1155/2014/647380

**Published:** 2014-05-15

**Authors:** Hai Wang, Yingfeng Cai, Long Chen

**Affiliations:** ^1^School of Automotive and Traffic Engineering, Jiangsu University, Zhenjiang 212013, China; ^2^Automotive Engineering Research Institute, Jiangsu University, Zhenjiang 212013, China

## Abstract

Vision based vehicle detection is a critical technology that plays an important role in not only vehicle active safety but also road video surveillance application. Traditional shallow model based vehicle detection algorithm still cannot meet the requirement of accurate vehicle detection in these applications. In this work, a novel deep learning based vehicle detection algorithm with 2D deep belief network (2D-DBN) is proposed. In the algorithm, the proposed 2D-DBN architecture uses second-order planes instead of first-order vector as input and uses bilinear projection for retaining discriminative information so as to determine the size of the deep architecture which enhances the success rate of vehicle detection. On-road experimental results demonstrate that the algorithm performs better than state-of-the-art vehicle detection algorithm in testing data sets.

## 1. Introduction 

Robust vision based vehicle detection on the road is to some extent a challenging problem since highways and urban and city roads are dynamic environment, in which the background and illuminations are dynamic and time variant. Besides, the shape, color, size, and appearance of vehicles are of high variability. To make this task even more difficult, the ego vehicle and target vehicles are generally in motion so that the size and location of target vehicles mapped to the image are diverse.

Although deep learning for object recognition has been an area of great interest in the machine-learning community, no prior research study has been reported that uses deep learning to establish an on-road vehicle detection method. In this paper, a 2D-DBN based vehicle detection algorithm is proposed.

The main novelty and contribution of this work include the following. (1) A deep learning architecture of 2D-DBN which preserves discriminative information for vehicle detection is proposed. (2) A deep learning based on-road vehicle detection system has been implemented and thorough quantitative performance analysis has been presented.

The rest of this paper is organized as follows.  Section 2 will give a brief talking about vision based vehicle detection tasks and deep learning for object recognition.  Section 3 introduces in detail the proposed 2D-DBN architecture and training methods for the vehicle detection tasks. The experiments and analysis will be given in Section 4 and Section 5 is the conclusion.

## 2. Related Research

In this section, a brief overview of two categories of work that is relevant to our research is introduced. The first set is about vision based vehicle detection and the second focuses on deep learning for object recognition.

### 2.1. Vision Based Vehicle Detection

Since only monocular visual perception is used in our project, this section will mainly refer to studies using monocular vision for on-road vehicle detection.

For monocular vision based vehicle detection, using vehicle appearance characteristics is the most common and effective approach. A variety of appearance features have been used in the field to detect vehicles. Some typical image features representing intuitive vehicle appearance information, such as local symmetry, edge, and cast shadow, have been used by many earlier works.

In recent years, there has been a transition from simpler image features to general and robust feature sets for vehicle detection. These feature sets, now common in the computer vision literature, allow for direct classification and detection of objects in images. For vehicle detection purpose, histogram of oriented gradient (HOG) features and Haar-like features are extremely well represented in literature. Besides, Gabor features, scale-invariant feature transform (SIFT) features, speeded up robust features (SURF), and some combined features are also applied for vehicle image representation.

Classification methods for appearance-based vehicle detection have also followed the general trends in the computer vision and machine learning literature. Compared to generative classifiers, discriminative classifiers, which learn a decision boundary between two classes (vehicles and unvehicles), have been more widely used in vehicle detection application. Support vector machines (SVM) and Adaboost are the most two common classifiers that are used for training vehicle detector. In [1], SVM classification was used to classify Haar feature vectors. The combination of HOG features and SVM classification has also been used [2–4]. Adaboost [5] has also been widely used for classification for Viola and Jones' contribution. The combination of Haar-like feature extraction and Adaboost classification has been used to detect rear faces of vehicles in [6–9]. Artificial neural network classifiers were also used for vehicle detection, but the training often failed due to local optimum [10].

### 2.2. Deep Learning for Object Recognition

Classifiers such as SVM and Adaboost referred to in last section are all indeed a shallow learning model because they both can be modeled as structure with one input layer, one hidden layer, and one output layer. Deep learning refers to a class of machine learning techniques, where hierarchical architectures are exploited for representation learning and pattern classification. Different from those shallow models, deep learning has the ability of learning multiple levels of representation and abstraction that helps to make sense of image data. From another point of view, deep learning can be viewed as one kind of multilayer neural networks with adding a novel unsurprised pretraining process.

There are various subclasses of deep architecture. Deep belief networks (DBN) modal is a typical deep learning structure which is first proposed by Hinton et al. [11]. The original DBN has demonstrated its success in simple image classification tasks of MNIST. In [12], a modified DBN is developed in which a Boltzmann machine is used on the top layer. This modified DBN is used in a 3D object recognition task.

Deep convolutional neural network (DCNN) with the ability to preserve the space structure and resistance to small variations in the images is used in image classification [13]. Recently, DCNN achieved the best performance compared to other state-of-the-art methods in the 2012 ImageNet LSVRC contest containing 1.2 million images with more than 1000 different classes. In this DCNN application, a very large architecture is built with more than 600,000 neurons and over 60 million weights.

**Figure 1:**
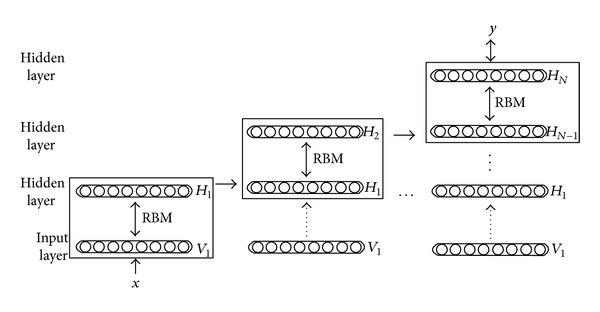
Architecture of deep belief network (DBN).

DBN is a probabilistic model composed of multiple layers of stochastic, hidden variables. The learning procedure of DBN can be divided into two stages: generative learning to abstract information layer by layer with unlabelled samples firstly and then discriminative learning to fine-tune the whole deep network with labeled samples to the ultimate learning target [11].  Figure 1 shows a typical DBN with one input layer *V*_1_ and* N* hidden layers *H*_1_, *H*_2_,…, *H*_*N*_, while* x* is the input data which can be, for example, a vector, and* y* is the learning target, for example, class labels. In the unsupervised stage of DBN training processes, each pair of layers grouped together to reconstruct the input of the layer from the output. In Figure 1, the layer-wise reconstruction happens between *V*_1_ and *H*_1_, *H*_1_ and *H*_2_,…, *H*_*N*−1_ and *H*_*n*_, respectively, which is implemented by a family of restricted Boltzmann machines (RBMs) [14]. After the greedy unsupervised learning of each pair of layers, the features are progressively combined from loose low-level representations into more compact high-level representations. In the supervised stage, the whole deep network is then refined using a contrastive version of the “wake-sleep” algorithm via a global gradient-based optimization strategy.

## 3. Deep Learning Based Vehicle Detection

In this section, a novel algorithm based on deep belief network (DBN) is proposed. Traditional DBN for object classification has some shortages. First, the training samples are regularized to first-order vector which will lead to the missing of spatial information contained by the image samples. This will obviously lead to a decline in the detection rate in vehicle detection tasks. Secondly, the size of layers (such as node number and layer number) in the traditional DBN is manually set which is often big and will lead to structural redundancy and increase the training and decision time of the classifier, while, for vehicle detection algorithm which is usually used in real-time application, decision time is a critical factor. The proposed 2D-DBN architecture for vehicle detection uses second-order planes instead of first-order vector of 1D-DBN as input and uses bilinear projection for retaining discriminative information so as to determine the size of the deep architecture. The bilinear projection maps original second-order output of lower layer to a small bilinear space without reducing discriminative information. And the size of the upper layer is that of the bilinear space.

In Section 3.1, the overall architecture of our 2D-DBN for vehicle detection will be introduced. In Section 3.2, the bilinear projection method of lower layer output will be given. In Sections 3.3 and 3.4, the training method of the whole 2D-DBN for vehicle detection will be deduced.

### 3.1. 2*D* Deep Belief Network (2D-DBN) for Vehicle Detection

Let *X* be the set of data samples including vehicle images and nonvehicle images, assuming that *X* is consisting with* K* samples which is shown below:

(1) X=[X1,X2,…,Xk,…,XK].

In *X*, **X**_*k*_ is training samples and in the image space **R** ^*I*×*J*^. Meanwhile, *Y* means the labels corresponding to *X*, which can be written as

(2) Y=[y1,y2,…,yk,…,yK].

In *Y*, *y*_*k*_ is the label vector of **X**_*k*_. If **X**_*k*_ belongs to vehicles, *y*_*k*_ = (1,0). On the contrary, *y*_*k*_ = (0,1).

The ultimate purpose in vehicle detection task is to learn a mapping function from training data *X* to the label data *Y* based on the given training set, so that this mapping function is able to classify unknown images between vehicle and nonvehicle.

**Figure 2:**
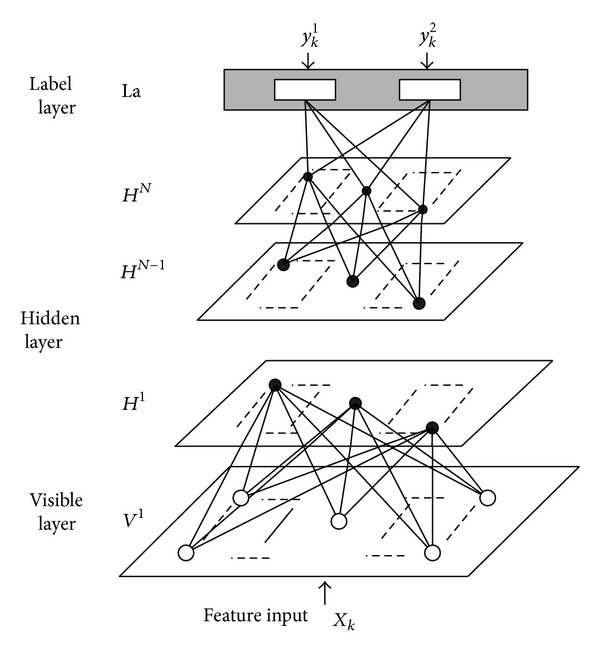
Proposed 2D-DBN for vehicle detection.

Based on the task described above, a novel 2D deep belief network (2D-DBN) is proposed to address this problem.  Figure 2 shows the overall architecture of 2D-DBN. A fully interconnected directed belief network includes one visible input layer *V* ^1^, *N* hidden layers *H* ^1^,…, *H* ^*N*^, and one visible label layer La at the top. The visible input layer *V* ^1^ maintains *M* × *N* neural and equal to the dimension of training feature which is the original 2D image pixel values of training samples in this application. Since maximum discriminative ability wishes to be preserved from layer to layer with nonredundant layer size in this application for real-time requirement, the size of *N* hidden layers is dynamically decided with so-called bilinear projection. In the top, the La layer just has two units which is equal to the classes this application would like to classify. Till now, the problem is formulated to search for the optimum parameter space *θ* of this 2D-DBN.

The main learning process of the proposed 2D-DBN has three steps.

1. The bilinear projection is utilized to map the lower layer output data onto subspace and optimized to optimum dimension as well as to retain discriminative information. The size of the upper layer will be determined by this optimum dimension.
2. When the size of the upper layer is determined, the parameters of the two adjacent layers will be refined with the greedy-wise reconstruction method. Repeat step (1) and step (2) till all the parameters of hidden layers are fixed. Here, step (1) and step (2) are so called pretraining process.
3. Finally, the whole 2D-DBN will be fine-tuned with the La layer information based on back propagation. Here, step (3) can be viewed as supervised training step.

### 3.2. Bilinear Projection for Upper Layer Size Determination

In this section, followed with Zhong's contribution [15], bilinear projection is used in order to determine the size of every upper layer in adjacent layer groups. As mentioned in Section 3.1, with the labeled training data **X**_*k*_ ∈ **R** ^*I*×*J*^ as the output of the visible layer *V* ^1^, bilinear projection maps the original data **X**_*k*_ onto a subspace and is represented by its latent form **L** **X**_*k*_. The bilinear projection is written as follows:

(3) LXk=UTXkV, k=1,2,…,K.

Here, **U** ∈ *R* ^*M*×*P*^ and **V** ∈ *R* ^*N*×*Q*^ are projection matrices that map the original data **X**_*k*_ by its latent form **L** **X**_*k*_ with the constraint that **U** ^*T*^ **U** = **I** and **V** ^*T*^ **V** = **I**.

How to determine the value of **U** and **V** so that the discriminative information of **X**_*k*_ can be preserved is the issue that needs to be solved. For this, a specific objective function is built as follows:

(4) argmax⁡U,V J(U,V)=∑i,j||UT(Xi−Xj)V||2×(αBij−(1−α)Wij),

in which *B*_*ij*_ is between-class weights, *W*_*ij*_ is within class weights, and *α* ∈ [0,1] is the balance parameter. *B*_*ij*_ and *W*_*ij*_ are calculated as follows [16]:

(5) Bij={−nnc(nnc+nc)nc,if  yic=yjc=11nnc+nc,else,Wij={1nc,if  yic=yjc=10,else.

Here, *y*_*i*_^*c*^ is the class label of sample data **X**_*i*_, which is either (1,0) or (0,1). *n*_*c*_ is the number of samples that belong to class *c* and *n*_*c*_ is those not belonging to class *c*. Since vehicle detection is a binary classification problem, *c* ∈ {1,2} in this application.

It can be seen that the purpose of the objective function is to simultaneously maximize the between-class distances and minimize the within-class distances. In other words, the objective function focuses on maximizing the discriminative information of all the sample data. However, optimizing *J*(**U**, **V**) is a nonconvex optimization problem with two matrices **U** and **V**. To deal with this trouble, a strategy called alternative fixing (AF) is used, which is fixing **U**  (or  **V**) and optimizing the objective function *J*(**U**, **V**) with just variable matrix **V** (or **U**) and then fixing **V** (or **U**) and optimizing *J*(**U**, **V**) with just **U** (or **V**). AF will be implemented alternatively till *J*(**U**, **V**) reaches its upper bound.

After the optimum process, new U* and V* that maximize *J*(**U**, **V**) are got and preserve the discriminative information of original sample data *X*. Based on this, then, the size of the upper layer can be determined by the number of positive eigenvalues of U* and V*, which is *P* and *Q*, respectively.

### 3.3. Pretraining with Greedy Layer-Wise Reconstruction Method

In last subsection, the size of the upper layer is determined to be *P* × *Q*. In this subsection, the parameters of the two adjacent layers will be refined with the greedy-wise reconstruction method proposed by Hinton et al. [11]. To illustrate this pretraining process, we take the visible input layer *V* ^1^ and the first hidden layer *H* ^1^ for example.

The visible input layer *V* ^1^ and the first hidden layer *H* ^1^ contract a restrict Boltzmann machine (RBM). *I* × *J* is the neural number in *V* ^1^ and *P* × *Q* is that of *H* ^1^. The energy of the state (*v* ^1^,*h* ^1^) in this RBM is

(6) E(v1,h1,θ1)=−(v1Ah1+b1v1+c1h1)=−∑i=1,j=1i≤I,j≤J∑p=1,q=1p≤P,q≤Qvij1Aij,pq1hpq1 −∑i=1,j=1i≤I,j≤Jbij1vij1−∑p=1,q=1p≤P,q≤Qcpq1hpq1,

in which *θ* ^1^ = (**A** ^1^, **b** ^1^, **c** ^1^) are the parameters between the visible input layer *V* ^1^ and the first hidden layer *H* ^1^. *A*_*ij*,*pq*_^1^ is the symmetric weights from input neural (*i*, *j*) in *V* ^1^ to the hidden neural (*p*, *q*) in *H* ^1^. *b*_*ij*_^1^ and *c*_*pq*_^1^ are the (*i*, *j*)^*th*^ and (*p*, *q*)^*th*^ bias of *V* ^1^ and *H* ^1^. So this RBM is with the joint distribution as follows:

(7) P(v1,h1;θ1)=1Ze−E(v1,h1;θ1)=e−E(v1,h1;θ1)∑v1∑h1e−E(v1,h1;θ1).

Here, *Z* is the normalization parameter and the probability that **v** ^1^ is assigned to *V* ^1^ of this modal is

(8) P(v1)=1Z∑h1e−E(v1,h1;θ1)=∑h1e−E(v1,h1;θ1)∑v1∑h1e−E(v1,h1;θ1).

After that, the conditional distributions over visible input state **v** ^1^ in layer *V* ^1^ and hidden state *h* ^1^ in *H* ^1^ are able to be given by the logistic function, respectively,

(9) p(h1 ∣ v1)=∏p,qp(hpq1 ∣ v1),p(hpq1 ∣ v1)=σ(∑i=1,j=1i≤I,j≤Jvij1Aij,pq1+cpq1),

(10) p(v1 ∣ h1)=∏i,jp(vij1 ∣ h1),p(vij1 ∣ h1)=σ(∑p=1,q=1p≤P,q≤Qhpq1Aij,pq1+bij1).

Here, *σ*(*x*) = 1/(1 + exp⁡(−*x*)).

At last, the weights and biases are able to be updated step by step from random Gaussian distribution values *A*_*ij*,*pq*_^1^(0),  *b*_*ij*_^1^(0), and *c*_*pq*_^1^(0) with Contrastive Divergence algorithm [17], and the updating formulations are

(11) Aij,pq1=ϑAij,pq1+εA(〈vij1(0)hij1(0)〉data−〈vij1(t)hij1(t)〉recon),bij1=ϑbij1+εb(vij1(0)−vij1(t)),cpq1=ϑcpq1+εc(hpq1(0)−hpq1(t)),

in which 〈·〉_data_ means the expectation with respect to the data distribution and 〈·〉_recon_ means the reconstruction distribution after one step. Meanwhile, *t* is step size which is set to *t* = 1 typically.

Above, the pretraining process is demonstrated by taking the visible input layer *V* ^1^ and the first hidden layer *H* ^1^ for example. Indeed, the whole pretraining process will be taken from low layer groups (*V* ^1^, *H* ^1^) to up layer groups (*H* ^*n*−1^, *H* ^*n*^) one by one.

### 3.4. Global Fine-Tuning

In the above unsurprised pretraining process, the greedy layer-wise algorithm is used to learn the 2D-DBN parameters with the information added from bilinear projection. In this subsection, a traditional back propagation algorithm will be used to fine-tune the parameters *θ* = [**A**, **b**, **c**] with the information of label layer La.

Since good parameters initiation has been maintained in the pretraining process, back propagation is just utilized to finely adjust the parameters so that local optimum parameters θ* = [A*, b*, c*] can be got. In this stage, the learning objection is to minimize the classification error $\left[-\sum_{t}y_{t}\log\;{\hat{y}}_{t}\right]$, where **y**_*t*_ and ${\hat{y}}_{t}$ are the real label and output label of data **X**_*t*_ in layer *N*.

## 4. Experiment and Analysis

**Figure 3:**
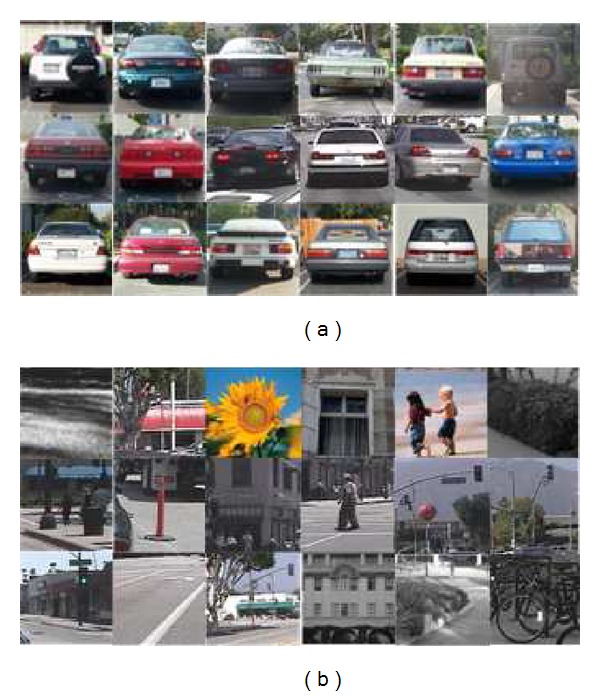
Some positive and negative training samples. (a) Positive samples. (b) Negative samples.

This section will take experiments on vehicle datasets to demonstrate the performance of the proposed 2D-DBN. The datasets for training are Caltech1999 database which includes images containing 126 rear view vehicles. Besides, another 600 vehicles in images are collected by our groups in recorded road videos for training. Meanwhile, the negative samples are chosen from 500 images not containing vehicles and the number of negative samples for training is 5000.  Figure 3 shows some of these positive and negative training samples. The testing datasets are recorded road videos with 735 manual marked vehicles.

By using the proposed method, three different architectures of 2D-DBN are applied. They all contain one visible layer and one label layer, but with one, two, and three hidden layers, respectively. In training, the critical parameters of the proposed 2D-DBN in experiments are set as *α* = 0.5 and *ϑ* = 0.8 and image samples for training are all resized to 32 × 32.

The detection results of these three architectures of 2D-DBN are shown in Table 1. It can be seen that 2D-DBN with two hidden layers maintains the highest detection rate.

**Figure 4:**
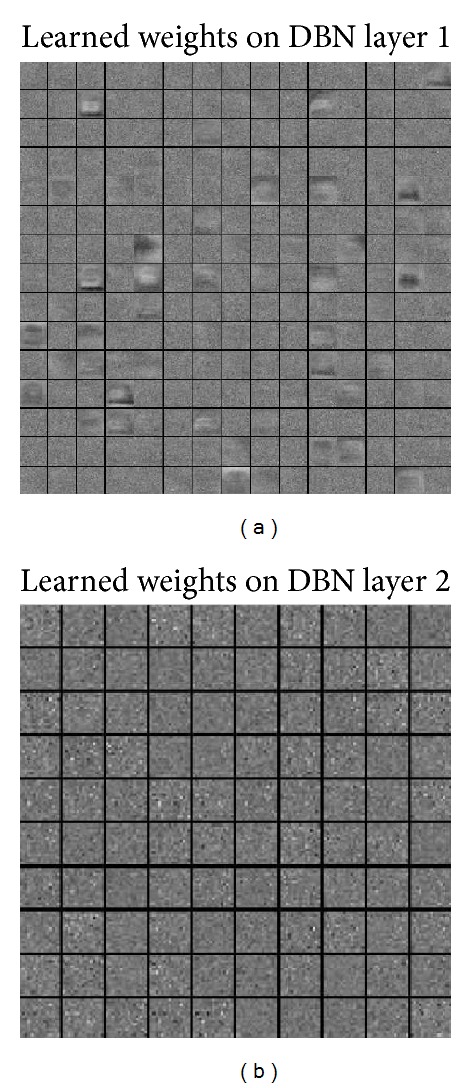
Learned weights of first hidden layer and second hidden layer on 2D-DBN: (a) weights of first hidden layer and (b) weights of second hidden layer.

The learned weights of hidden layers are shown in Figure 4.

Then, we compared the performance of our 2D-DBN with many other state-of-the-art classifiers, including support vector machine (SVM),* k*-nearest neighbor (KNN), neural networks, 1D-DBN, and deep convoluted neural network (DCNN).

The detection results of these methods are shown in Table 2.

From the compared results, it can be concluded that classification methods with deep architecture, for example, 1D-DBN, DCNN, and 2D-DBN are significantly better than those of shallow architecture, for example, SVM, KNN, and NN. Moreover, our proposed 2D-DBN is better than 1D-DBN and DCNN due to 2D feature input and the bilinear projection.

**Figure 5:**
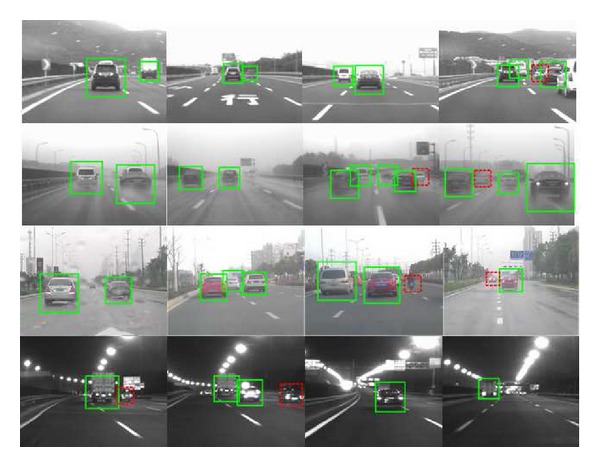
Some of the real road vehicle sensing results. First row: daylight highway situation; second row: raining day highway situation; third row: daylight urban situation; forth row: night highway with road lamp.

Finally, this 2D-DBN vehicle detection method is utilized on road vehicle detection system and some of the vehicle sensing results in real road situation are shown in Figure 5. The four rows of images are picked in daylight highway, raining day highway, daylight urban, and night highway with road lamp, respectively. The solid green box means detected vehicles, and the dotted red box means undetected vehicles or false detected vehicles. The average vehicle detection time for one frame with 640 × 480 resolution is around 53 ms in our Advantech industrial computer.

Overall, most of the on-road vehicles can be sensed successfully while misdetection and false detection sometimes occurred during adverse situations such as partial occlusion and bad weather.

## 5. Conclusion 

In this work, a novel vehicle detection algorithm based on 2D-DBN is proposed. In the algorithm, the proposed 2D-DBN architecture uses second-order planes instead of first-order vector as input and uses bilinear projection for retaining discriminative information so as to determine the size of the deep architecture which enhances the success rate of vehicle detection. On-road experimental results demonstrate that the system works well in different roads, weather, and lighting conditions.

The future work of our research will focus on the situation when a vehicle is partially occluded with deep architecture framework.

## Figures and Tables

**Table 1:** Detection results of three different architectures of 2D-DBN.

Classifier types	Correct labeling	Correct rate
2D-DBN (1H)	689/735	93.74%

**2D-DBN (2H)**	**706/735**	**96.05%**
2D-DBN (3H)	695/735	94.56%

**Table 2:** Detection results of multiple methods.

Classifier types	Correct labeling	Correct rate
SVM	658/735	89.52%
KNN	642/735	87.35%
NN	619/735	84.21%
1D-DBN	684/735	93.06%
DCNN	697/735	94.83%
**2D-DBN (2H)**	**706/735**	**96.05%**
